# Supplementary figures and images for: Expression and purification of human diacylglycerol kinase α from baculovirus-infected insect cells for structural studies
Source: PeerJ. 2018 Aug 10;6:e5449. doi: 10.7717/peerj.5449 (PMC6089211; doi:10.7717/peerj.5449)

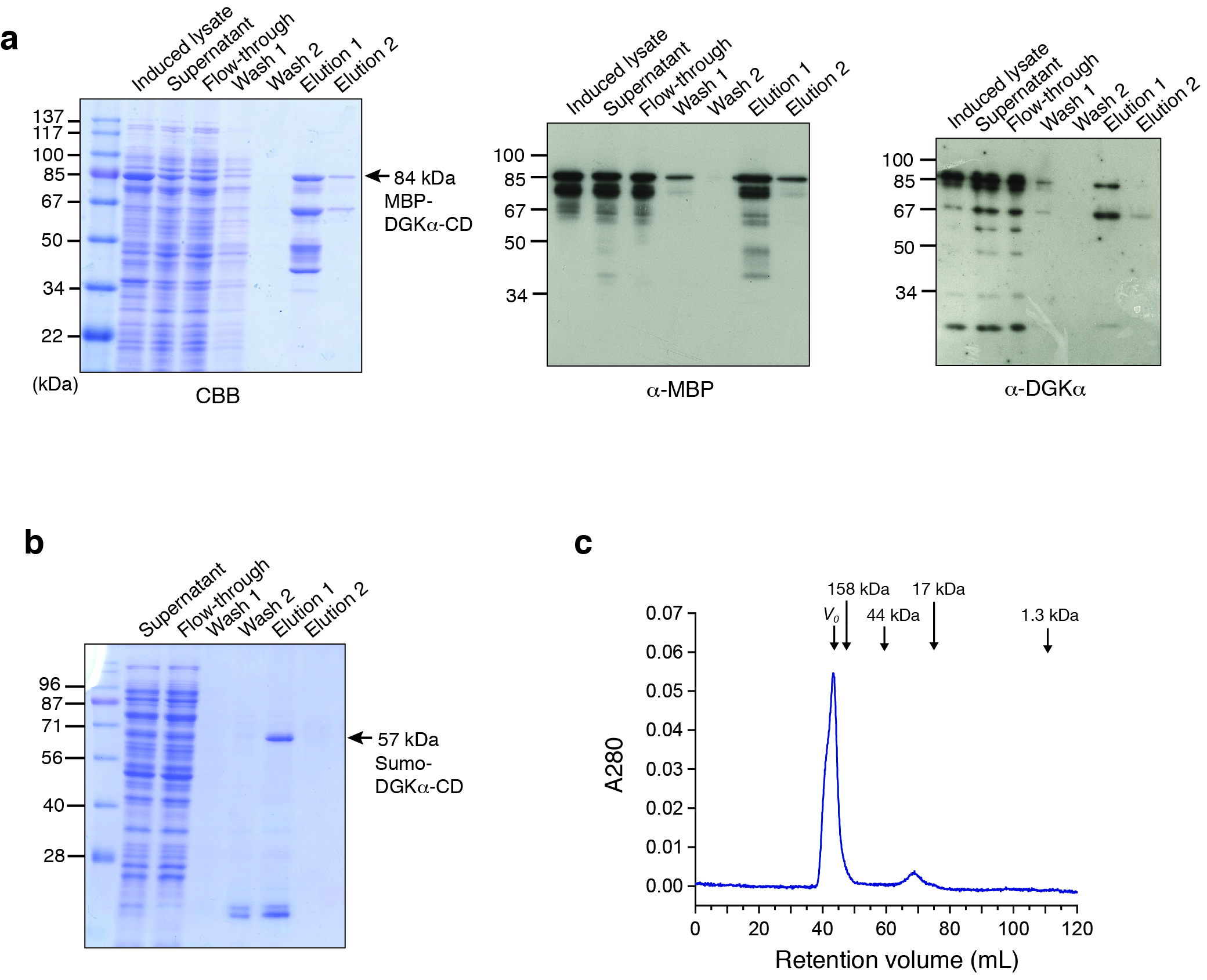

Supplement: Supplemental Information 1 — (A) SDS-PAGE (10%) analysis of the amylose-affinity purification of MBP-fused DGKα-CD (D369–S735). Left, Coomassie brilliant blue staining. In elution fractions, proteins were eluted with 10 mM maltose. The total mass of MBP-DGKα-CD is 84.1 kDa and the corresponding band is indicated by an arrow. Middle and right, the immunoblot analysis using anti-MBP antibody (middle) and anti-DGKα antibody (right). In addition to the band of MBP-DGKα-CD, several smaller mass bands were reactive to those two antibodies. (B) After Ni-affinity purification of Hisx6-Sumo-DGKα-CD, the samples were analyzed with SDS-PAGE (12%) followed by CBB staining. Bound proteins were eluted with 300 mM imidazole. The total mass of Hisx6-Sumo-DGKα-CD (D344–S735EE) is 57 kDa. (C) Elution profile of the purified Sumo-fused DGKα-CD from Superdex 75 16/60 column. Void volume (V0) and the elution volume of the standard molecules, γ-globulin (158 kDa), ovalbumin (44 kDa), myoglobin (17 kDa), and vitamin B12 (1.3 kDa) are indicated by arrows. Most of proteins eluted in a void volume of the column (43 mL). Very small peak corresponding to a molecular mass of 20 kDa was also appeared. [file peerj-06-5449-s001.jpg]

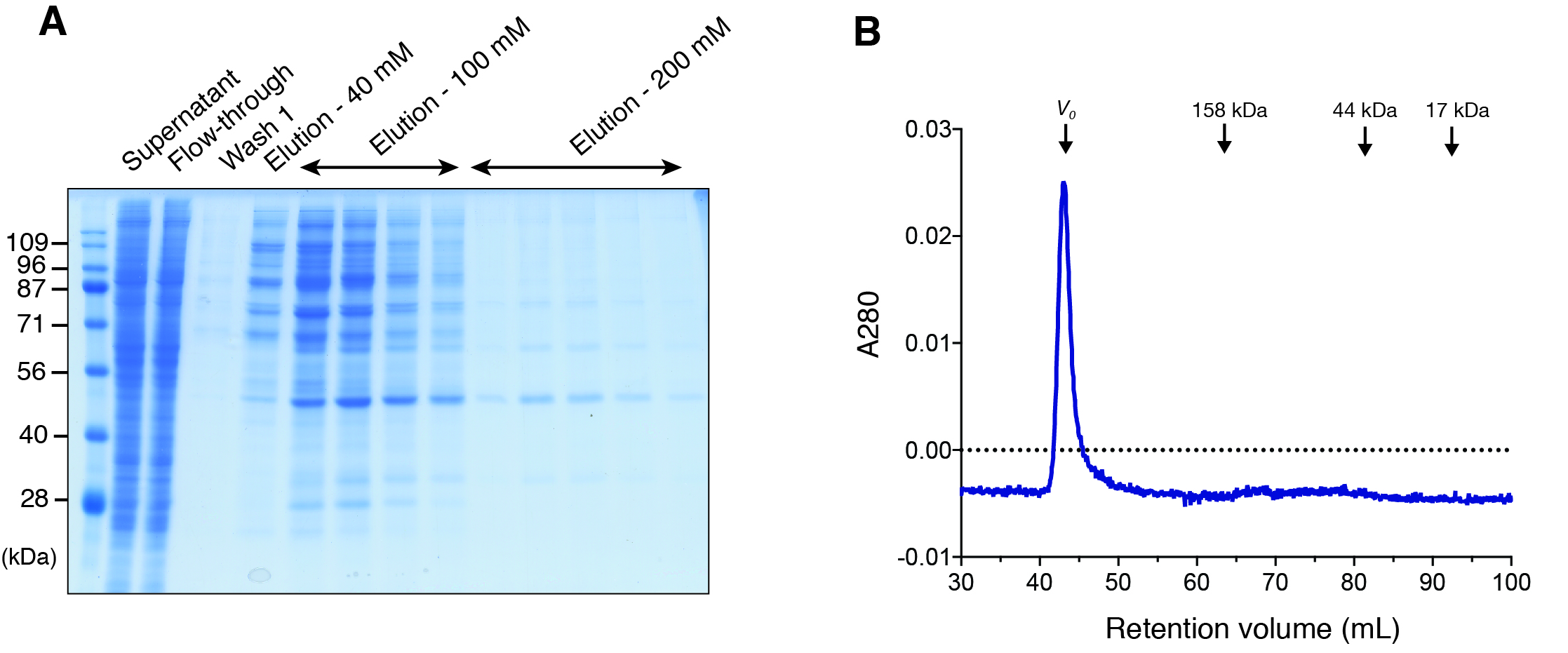

Supplement: Supplemental Information 2 — (A) SDS-PAGE (10%) analysis of fractions from Ni2+-affinity purification of Hisx6-DGKα-CD (D364–S735) expressed in Sf9 cells. Elution from the column was done with 40 mM, 100 mM, and 200 mM imidazole. The total mass of the DGKα-CD construct is 44.1 kDa. (B) Elution fractions with 200 mM imidazole from Ni2+-affinity chromatography was analyzed by a size-exclusion chromatography on a Superdex 200 16/60 column equilibrated with 50 mM Tris-HCl, pH 7.5, 50 mM NaCl, 10% glycerol, and 3 mM DTT. DGKα-CD eluted in the void volume of the column. Void volume (V0) and the elution volume of the standard proteins, γ-globulin (158 kDa), ovalbumin (44 kDa), myoglobin (17 kDa) are indicated by arrows. [file peerj-06-5449-s002.jpg]

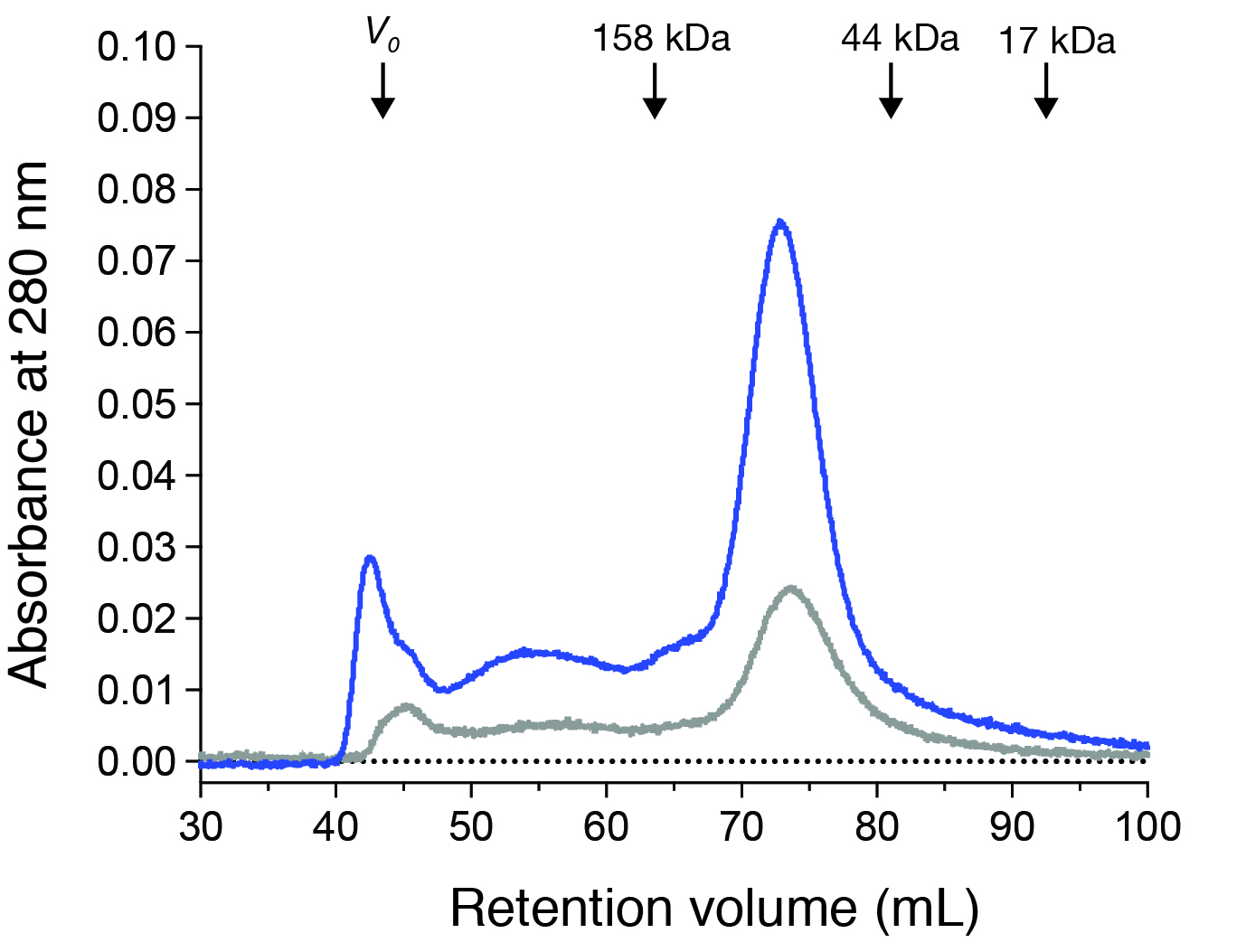

Supplement: Supplemental Information 3 — Fifteen milliliter of Elution fraction (50 mM imidazole) of DGKα in Ni2+affinity chromatography (Fig. 1C) was concentrated to 4 mL using a centrifugal filter (Amicon Ultra-15) and applied to a Superdex 200 16/60 column equilibrated with 20 mM Tris-HCl, pH 7.4, 200 mM NaCl, 3 mM CaCl2, 3 mM MgCl2, 0.5 mM DTT, 5 % glycerol. Elution profile of the concentrated sample was shown in blue color and that of the sample before concentration (Fig. 1D) was shown in gray for comparison. Void volume (V0) and the elution volume of the standard proteins, γ-globulin (158 kDa), ovalbumin (44 kDa), myoglobin (17 kDa) are indicated by arrows. [file peerj-06-5449-s003.jpg]
